# Has the BMI had its day?

**DOI:** 10.1038/s41366-024-01643-y

**Published:** 2024-10-07

**Authors:** Manfred J. Müller, Anja Bosy-Westphal

**Affiliations:** Institute of Human Nutrition and Food Science, Christian-Albrechts-Universität zu Kiel, Kiel, Germany

**Keywords:** Health sciences, Epidemiology

BMI is an index of body weight corrected for body height calculated as kg/m^2^. In epidemiology, clinical practice, and research BMI is used for characterization and categorization of weight status. BMI is simple to calculate, it is documented in individual health records and is assumed to be applicable to every population. When compared with other weight-to-height indices, in adults BMI had the weakest correlation with height while BMI has the strongest correlation with fat mass (FM [1]). Nevertheless, a residual correlation with height persists. In addition, adiposity and sex decrease the power of height standardization of body weight [2]. In addition, different heights influence the prevalence of people with obesity: In adults, the prevalence of BMI > 30 kg/m^2^ gradually increased with decreasing percentile of height whereas in children and adolescents, a positive association between height and weight status was observed [3].

Since short and tall subjects with equivalent BMIs do not have an identical body composition, BMI cannot distinguish between FM and fat-free mass (FFM [4];). The variance in body composition in subjects with equivalent BMI may also explain that BMI better reflects fat mass and cardiometabolic risks in taller subjects compared with those of shorter statures [5]. Finally, the age- and sex-adjusted association between BMI and total body fat mass becomes stronger with increasing height [4] and FM scaled weakly with height [6]. Thus, different proportions of people with obesity within different populations limit (i) the comparison of the BMI of two populations and (ii) the use of BMI-reference data due to various degrees of height standardization of body weight (i.e., the scaling exponent varies depending on the position of the centile curve [6]).

In children, the optimal height power of body weight is still a matter of debate. Previous data indicated that at different ages values ranged between 1.0 and 2.5, with high values of 2.5 to 3.0 observed during puberty. There was considerable inter-individual variation, indicating an age-dependence of the scaling exponent [2]. Although BMI is known to provide an inaccurate weight standardization in children, the age- and sex-dependent 90^th^ (> + 1 SD; equivalent to BMI 25 kg/m^2^ at age 19 years) or 97^th^ BMI-percentiles (> + 2 SD; equivalent to BMI 30 kg/m^2^ at age 19 years) are still used as a proxy of childhood overweight and obesity [7].

In this issue of IJO, Hudda and colleagues [8] have assessed the optimal height powers in 391,801 children at ages of 7, 10 and 13 yrs and have analyzed their consistence between different birth year periods from 1930 to 1996 including the period of the obesity epidemic starting in the 70ties of the last century. The cross-sectional data from the Copenhagen School Health Records Register (CSHRR) indicated that the height powers of body weight was approximately 2.20 in young children, increasing with age (i.e., during puberty to 2.82 and 2.92 in boys and girls, respectively) and birth years falling back again in adolescence and during the obesity epidemic. Up to 9% of the variance in BMI was still explained by height. While the data set may be considered as historical and the inclusion of more recent data would have been beneficial, the present paper makes a significant contribution to our present knowledge as the data provide compelling evidence against (i) an uncritical use of the BMI in different populations of children and (ii) the general validity of the current weight status definition.

The results of the CSHRR contribute to existing longitudinal data addressing the meaning of the BMI in 49,717 individuals born in 1946, 1958, 1970 or 2001 across age and time (1956–2015) in England [2]. In that study, the changes over time in the obesogenic environment appear to have increased the height power of weight close to slightly above 2 in young children, reaching a value of >3 at age 10 and 11 (corresponding to a ‘tri-ponderal mass index’ of kg/m^3^ [9];). However, the height powers secondly fall back and stabilized at ≤2.0 in the older cohorts. Once more, the optimal power of height standardization of body weight takes different values in different groups and individuals [2].

The combined findings of the two studies show that (i) BMI is not consistently precise or objective means of normalization of body weight for height and (ii) a single index cannot be used for categorization of obesity in children and adolescents. The Benn Index (i.e., weight/height^p^, with p is population specific [9, 10];) or the use of an ‘extended’ allometric analysis (BMI_ext_) based on multi-scaling in weight-for-height distribution (i.e., weight/height^p*q*^, with p*q* provided by quantile regression [6];) are alternative methods eliminating the impact of height on BMI in children and adolescents.

Going beyond the BMI, Hudda et al. [8] have translated their body weight-to-height power estimates to a 2-compartment model of body composition thereby standardizing FM and FFM. Both, fat and FFM are normalized for height^2^ (as FMI, FFMI) as a means of adjusting their masses for between-subject differences in stature [11, 12]. In children, the optimal height powers required to standardize FM and FFM for height were found to be 2.46–4.27 and 2.12–2.86, respectively, with variations observed across birth cohorts, sexes, pubertal status and ethnic groups [8]. In younger children, there was an initial increase in FMI and (less pronounced) FFMI with birth cohorts, with girls exhibiting higher values than boys. However, there was a subsequent decrease in more recent decades. While in the CSHRR data set no direct measurements of body composition were available, FM and FFM were calculated based on weight and height using an algorithm which had been ‘validated’ against DXA- and BIA-estimates (which, both, are not gold-standard methods) in children from the Avon Longitudinal Study of Parents and Children (ALSPAC) with an estimation error of 2.6 kg FM [13]. Understandably, that body composition algorithm could not be validated retrospectively in the CSHRR-children. However, comparing the weight-to-height-power estimates the data suggest that FMI has the same limitations due to variations at both, the individual and the group level.

A 2-compartment model does not fully account for anatomical body composition. The masses of individual organs and tissues scale differently with weight and height [4, 14]. In adults with a BMI between 15 and 48 kg/m^2^, whole body MRI data reveal that the organ weight-to-height powers vary between 0.83 to 0.92 for the brain, 1.36 for the kidneys, 2.16 to 3.40 for skeletal muscle and 2.48 for bone mass. In contrast, these values are close to 2 for liver and heart suggesting that at least brain and bone do not scale with weight with powers approximating 2 [4, 14]. Using data obtained in 421 children and adolescents (taken from the whole-body MRI-data base of the German Reference Centre of Body Composition [15];) the optimal power of height standardization was around 2 to 3 for FFM, liver, heart, kidneys with significantly higher values for spleen and FM, while the *p*-value for brain was <1 (Table 1). A reanalysis of data in a subgroup of 121 pubescent children showed similar p-exponents. The inter-individual variance related to scaling of individual organ and tissues masses to height characterize constitution-related variances which may explain up to 40% of the inter-individual variances in metabolism [14]. Obviously, there is no unique height power of body weight, FM, FFM, individual organs and tissues questioning the idea of a unique scaling of masses to height.

**Table 1:** Optimal power for height-standardisation (**p**) of childhood body weight, fat mass, fat-free mass and individual organ masses.

		Girls			Boys	
	x ± *SD*	r^a^	*p*	x ± *SD*	r^a^	*p*
Height, m	1.58 ± 0.15		-	1.62 ± 0.18		-
Weight, kg	60.2 ± 24.5	0.85**	3.59	62.2 ± 28.3	0.89**	4.24
FM, kg	19.9 ± 15.2	0.68**	6.00	15.8 ± 15.6	0.56**	5.00
FFM, kg	40.3 ± 11.4	0.93**	3.18	46.4 ± 16.9	0.96**	3.36
Brain, kg	1.42 ± 0.13	0.21*	0.16	1.56 ± 0.14	0.39*	0.39
Liver, kg	1.16 ± 0.37	0.81**	2.60	1.26 ± 0.44	0.86**	3.23
Heart, kg	0.23 ± 0.07	0.85**	2.72	0.27 ± 0.09	0.85**	3.18
Kidneys, kg	0.19 ± 0.06	0.78**	2.65	0.20 ± 0.07	0.85**	3.18
Spleen, kg	0.19 ± 0.09	0.71**	3.85	0.23 ± 0.13	0.77**	4.41

Detailed body composition data were obtained in 221 girls and 200 boys, age range 6–18 years. Fat mass (FM) and fat-free mass (FFM) were measured by Air Displacement Plethysmography, individual organ masses by whole body Magnetic Resonance Imaging (MRI). For details of the study population and methods of body composition analysis see ref. [13, 14]. The optimal height scaling power to create an index of weight or FM or FFM or individual organ masses that are minimally correlated with height was developed as an index weight/height^p^ where *p* is equal to *p* = r x*(SD of log weight/SD of log height).

^a^r is the correlation coefficient between log-transformed weight (or organ and tissue masses) and log transformed height (acc. to ref. [7, 8]).

*p < 0.05, **p < 0.01.

Hudda et al. [8] have merit in prompting return to a fundamental question that has yet to be resolved. While BMI is generally available, it provides only a crude estimate of overweight and obesity without a true biological background where BMI categories are based on BMI-related health risks found in epidemiological studies only. BMI still has some value in clinical practice, i.e., it is part of a recent framework for the diagnosis, staging and management of people with obesity [16]. By contrast, its value in research on the causes and metabolic consequences of obesity is very limited. It could be argued that the limitations of BMI hindered the success of obesity research to date. We should consider this with an open mind. Future phenotyping of patients should focus on medical and functional domains rather than on weight categories.
